# Cognitive Impairment in Sjögren's Disease: Unmasking Alzheimer's Through CSF Biomarkers. A Case Report

**DOI:** 10.1002/ccr3.71909

**Published:** 2026-01-20

**Authors:** Eliza Georgiou, Ruth Comber, Mina Alemam, Lisa Crosby, Anna Kirwan, Fiona Smyth, Elaine Greene

**Affiliations:** ^1^ Memory Clinic, MISA, St James Hospital Dublin Ireland; ^2^ Faculty of Medicine, Trinity College Dublin Dublin Ireland; ^3^ Department of Psychiatry, Faculty of Medicine University of Patras Patras Greece

**Keywords:** Alzheimer's Disease, autoimmune, biomarkers, MCI, Sjögren's Disease

## Abstract

Cognitive impairment in Sjögren's disease (SD) is typically attributed to autoimmune mechanisms or small vessel disease. However, its overlap with neurodegenerative pathology remains poorly understood. We report a 74‐year‐old woman with clinically diagnosed SD presenting with progressive memory decline. She demonstrated severe amnestic deficits on neuropsychological testing and had a cerebrospinal fluid (CSF) profile consistent with Alzheimer's disease (AD), including low Aβ1–42 and elevated total tau and phosphorylated tau. MRI revealed only mild small vessel disease. This case highlights the diagnostic importance of CSF biomarkers in distinguishing neurodegenerative from autoimmune cognitive syndromes. Though the overlap may be coincidental, it raises the question of whether systemic inflammation in SD could contribute to AD pathogenesis. This case illustrates how neurodegenerative disease may be overlooked in patients with autoimmune conditions. In individuals with SD and persistent amnestic symptoms, biomarker testing can help distinguish Alzheimer's pathology from autoimmune cognitive syndromes and guide more accurate diagnosis and care.

## Key Clinical Message

In patients with clinically diagnosed Sjögren's disease and progressive cognitive decline, Alzheimer's pathology should be actively considered. CSF biomarker testing can help avoid diagnostic overshadowing by autoimmune mechanisms and guide more accurate diagnosis and care.

## 1 Introduction

Sjögren's Disease (SD) is a systemic autoimmune disorder primarily affecting exocrine glands, often leading to sicca symptoms, often manifesting with sicca symptoms such as xerostomia and ocular dryness [1]. However, extraglandular manifestations, including central nervous system involvement and cognitive impairment, are increasingly recognized [2]. Cognitive impairment in SD is not uncommon, typically presenting as mild and fluctuating deficits attributed to autoimmune encephalopathy, small vessel disease, or chronic inflammation [3, 4, 5, 6]. However, the relationship between SS and neurodegenerative diseases such as Alzheimer's disease (AD) remains poorly understood. Several case reports have described dementia‐like syndromes in SD [7, 8, 9] but very few have explored the role of AD pathology through cerebrospinal fluid (CSF) biomarker analysis.

Here, we report the case of a 74‐year‐old woman with recently diagnosed SD who presented with progressive memory impairment and a CSF profile consistent with AD. To our knowledge, this is among the first reported documented cases of a biomarker‐confirmed amnestic mild cognitive impairment (MCI) on the Alzheimer's continuum in a patient with SD, raising important questions about coincidence versus potential mechanistic overlap.

## 2 Case History/Examination

The patient, a 74‐year‐old woman, was referred to the memory clinic following 2 years of progressive short‐term memory loss, word‐finding difficulty, and occasional temporal disorientation. Symptoms began shortly after a clinical diagnosis of SD made by a rheumatologist based on sicca symptoms, namely keratoconjunctivitis sicca (confirmed by optician), xerostomia, and compatible systemic findings. Serological data (e.g., anti‐SS‐A/SS‐B antibodies) and salivary gland biopsy results were not available at the time of writing. The patient was prescribed symptomatic treatment including artificial tears and oral pilocarpine.

At the time of referral, her GP reported concerns about increasing forgetfulness, repetitive questioning, and difficulties with managing appointments and daily routines. Initial cognitive screening in primary care showed a Montreal Cognitive Assessment (MoCA) [10] score of 26/30 and a Mini‐Mental State Examination (MMSE) [11] score of 25/30, both indicating mild impairments in delayed recall and visuospatial domains. The patient's score on the Alzheimer's Questionnaire (AQ) was 9/27, a brief 27‐item informant‐based screening tool in which scores ≥ 5 are considered suggestive of cognitive impairment [12].

Despite these concerns, the patient remained functionally independent. She continued driving and actively participated in weekly bridge games. Her husband observed increasing repetition and difficulty with scheduling and planning.

Her past medical history included hypothyroidism, hyperlipidemia, fibromyalgia, osteoarthritis, and remote rectal cancer resection. There was no known history of psychiatric or neurological illness. Her medication list included levothyroxine, rosuvastatin, aspirin, amlodipine, fesoterodine (antimuscarinic), nebivolol, cholecalciferol (vitamin D3), and lansoprazole. Notably, fesoterodine has been associated with potential anticholinergic cognitive effects, although its relevance to the patient's profile was deemed minimal given the biomarker and neuropsychological findings (see Discussion).

On clinical examination, the patient appeared well‐groomed and articulate, with normal orientation and no evidence of mood or behavioral disturbance. Neurological examination revealed no signs of parkinsonism, ataxia, or gait instability, aside from a mild postural tilt related to longstanding knee osteoarthritis.

Her cognitive screening battery included the Addenbrooke's Cognitive Examination—Revised (ACE‐R) [13] and the Repeatable Battery for the Assessment of Neuropsychological Status—Update (RBANS‐U) [14], both of which supported a diagnosis of amnestic mild cognitive impairment. RBANS‐U results are shown in Table 1.

**TABLE 1:** RBANS‐U (repeatable battery for the assessment of neuropsychological status).

RBANS‐U	Index score	Percentile
Immediate memory	49	< 0.1
Visuospatial/constructional	102	55th
Language	71	3rd
Attention	109	73rd
Delayed memory	52	0.1

Detailed cognitive testing confirmed the presence of an amnestic profile. Her ACE‐R/III score was 79/100, below the typical cut‐off of 82. Subdomain scores were as follows: Attention and Orientation 15/18, Memory 14/26, Verbal Fluency 9/14, Language 26/26, and Visuospatial Abilities 15/16.

On the RBANS‐U, significant deficits were observed in both immediate and delayed memory and language (Table 1), while attention and visuospatial functioning remained preserved.

## 3 Differential Diagnosis, Investigations and Treatment

**FIGURE 1:**
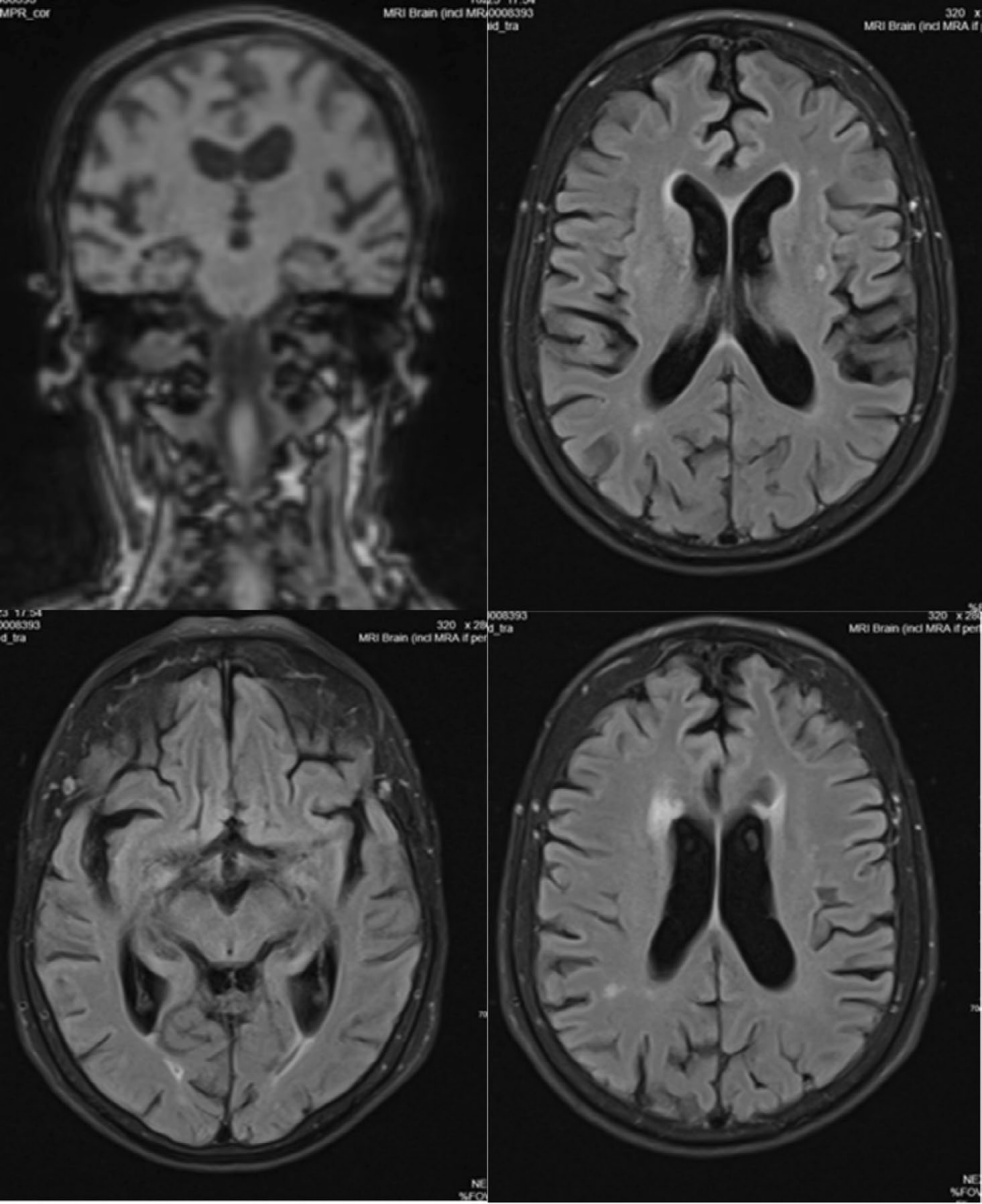
MRI brain‐fluid‐attenuated inversion recovery (FLAIR) sequences (showing periventricular WMHs).

Magnetic Resonance Imaging (MRI) of the brain demonstrated mild periventricular and subcortical white matter hyperintensities, consistent with small vessel disease. There was no hippocampal atrophy or evidence of inflammatory change. See Figure 1.

A comprehensive occupational therapy assessment revealed preserved independence in personal and instrumental activities of daily living, supported by environmental adaptations and spousal assistance. Functional assessment using the Kettle Test [15] (score: 11/52) suggested challenges in novel task execution and multi‐step processing. The Performance Assessment of Self‐Care Skills (PASS) [16] indicated mild difficulty in medication management, particularly with retention of complex instructions, though physical safety remained intact. The patient demonstrated good task learning potential but relied on structured support, environmental cues, and routine familiarity to maintain independence. These findings reflect a mild functional cognitive impairment consistent with her neuropsychological and biomarker profile.

Basic blood work including vitamin B12, thyroid function, folate, and CRP were within normal limits. Mild hyponatremia was observed (Na^+^ = 133 mmol/L), without clinical signs of dehydration or systemic illness, with no systemic inflammation or cardiac abnormalities on ECG. The CSF was clear and colorless, with normal opening pressure, acellular content (0 WBCs, 0 RBCs), and normal glucose and protein levels (protein: 0.42 g/L). Culture was negative for bacterial growth. Taurine was mildly elevated at 634 pg/mmol, a finding that, while nonspecific, has been noted in various neuroinflammatory and neurodegenerative states due to its osmoregulatory and neuroprotective roles. However, its clinical significance remains uncertain in this context. Importantly, autoimmune encephalitis markers were not tested, but the absence of pleocytosis, elevated protein, or radiological signs of inflammation makes autoimmune CNS involvement unlikely. In contrast, the CSF AD biomarker profile (low Aβ1–42: 441.9 pg/mL; and elevated phosphorylated tau: 115.1 pg/mL; p‐tau/Aβ42 ratio: 0.26) strongly supports a diagnosis on the Alzheimer's continuum per NIA‐AA criteria (Table 2) [17], providing a clear pathophysiological explanation for the patient's memory syndrome.

**TABLE 2:** Cerebrospinal fluid biomarkers.

CSF biomarkers		
Biomarker	Result	Interpretation
Amyloid β1–42	441.9 pg/mL	Normal range (591.0–997.0) Low
Taurine	634.0 pg/mL	Normal range (135.0–345.0) Elevated
Phosphorylated tau (p‐tau)	115.1 pg/mL	Normal range (35.0–64.0) Elevated
p‐tau/Aβ42 ratio	0.26	Normal < 0.067. Consistent with AD pathophysiology

## 4 Conclusion and Results

A multidisciplinary team diagnosis of amnestic mild cognitive impairment due to AD was reached, incorporating neuropsychological, functional, imaging, and biomarker data. The patient and her husband were invited for a structured feedback session, jointly led by the clinician and memory service social worker. Pharmacological treatment with an acetylcholinesterase inhibitor was discussed but deferred pending symptom progression and further follow‐up.

Patient was referred and participated in the EverActive programme, a community‐based initiative combining brain health education with a balance and stability exercise class. Conducted by a Postural Stability Instructor and Clinical Specialist Physiotherapist, the programme integrates elements from the Falls Management Exercise (FaME) and Otago Falls programmes [17, 18]. Exercises are tailored to individual needs, with a focus on strength, conditioning, and fall prevention. The patient attended twice within a seven‐week period. Due to pre‐existing joint pain, a seated exercise adaptation was recommended. While she required regular prompting and visual demonstrations to follow instructions, she remained actively engaged with the group and interacted well with both instructors and peers.

## 5 Discussion

Cognitive impairment is a well‐recognized extraglandular manifestation of SD, commonly attributed to mechanisms such as autoimmune encephalopathy, cerebral small vessel disease, or systemic fatigue‐related dysfunction—colloquially referred to as “brain fog” [4, 5, 19] True neurodegenerative pathology, particularly Alzheimer's disease, has not been clearly demonstrated with biomarker evidence in this population.

This case is, to our knowledge, the first to document a CSF biomarker profile meeting NIA‐AA criteria for Alzheimer's continuum (low Aβ42 and elevated tau/p‐tau) in a patient with clinically diagnosed SD and amnestic MCI [20, 21]. While the co‐occurrence may reflect age‐related risk, the temporal proximity between the SD diagnosis and the onset of progressive amnestic symptoms raises the possibility of an underlying pathophysiological interplay.

Emerging literature suggests that chronic systemic inflammation and immune dysregulation may contribute to AD development through mechanisms such as impaired β‐amyloid clearance, microglial overactivation, and persistent cytokine release [6, 21, 22, 23]. These processes may not initiate Alzheimer's pathology independently, but they may serve as accelerants in individuals with underlying vulnerability, whether genetic, metabolic, or cognitive.

In contrast to previously published reports of dementia in SD, which often involve autoimmune encephalitis or rapidly progressive syndromes responsive to immunotherapy [7, 8, 9]. Our patient presented with a typical AD pattern: insidious onset, amnestic‐predominant deficits, normal inflammatory markers, and no radiographic signs of encephalitis. Her profile—characterized by impaired encoding and delayed recall with preserved attention and executive function—is aligned with early‐stage AD rather than autoimmune‐mediated cognitive dysfunction. Additionally, the patient was prescribed fesoterodine, an antimuscarinic agent used for overactive bladder, which has been associated with anticholinergic burden and potential cognitive side effects in older adults, particularly in those at risk for neurodegeneration [24, 25].

While biomarker‐confirmed AD in SD is rarely reported, the broader spectrum of autoimmune connective tissue diseases—including systemic lupus erythematosus, rheumatoid arthritis, and systemic sclerosis—has demonstrated associations with reduced cognitive performance and increased psychiatric burden. For example, in a recent study of patients with rheumatoid arthritis and systemic sclerosis, reduced cognitive performance and high rates of depressive symptoms were observed, particularly affecting attention and executive domains [26]. Though the mechanisms may differ, such findings support the broader hypothesis that chronic inflammation and autoimmunity can contribute to neurocognitive vulnerability, and underscore the need for more targeted biomarker‐based research in conditions like SD.

Emerging epidemiological data suggest that patients with autoimmune diseases, including SD, may have an increased long‐term risk of developing Alzheimer's disease [6]. However, biomarker‐based studies remain limited. Notably, one small study by Dehlin et al. found reduced CSF Aβ42 levels in a subset of SS patients, suggesting that AD‐like pathology may exist in some cases but go undetected without targeted biomarker assessment [27].

This case highlights the need for careful cognitive screening and biomarker assessment in autoimmune patients presenting with cognitive symptoms, particularly when symptoms are slowly progressive, focal, and unaccompanied by systemic inflammatory flares.

A key limitation of this report is the absence of confirmatory serologic markers or salivary gland biopsy results to support the diagnosis of SD. While the diagnosis was made by a rheumatologist and the patient reported longstanding sicca symptoms, we acknowledge that alternative causes—particularly age‐related changes and polypharmacy—can also contribute to sicca presentations in older adults. As such, this case should be interpreted as illustrating the importance of not attributing amnestic symptoms solely to presumed autoimmune mechanisms, especially when biomarker‐based evidence of neurodegeneration is available.

This case underscores the importance of comprehensive cognitive assessment in patients with SD presenting with memory complaints. While cognitive symptoms in autoimmune conditions are often attributed to inflammatory or vascular mechanisms, this report highlights that coexisting AD may go unrecognized without formal biomarker evaluation. In the absence of systemic inflammatory signs or rapid progression, clinicians should maintain a high index of suspicion for neurodegenerative pathology and consider CSF analysis where appropriate to guide diagnosis, prognosis, and care planning.

## Author Contributions

**Eliza Georgiou:** conceptualization, investigation, writing – original draft, writing – review and editing. **Ruth Comber:** resources, writing – original draft, writing – review and editing. **Mina Alemam:** resources, writing – original draft, writing – review and editing. **Lisa Crosby:** investigation, methodology, writing – review and editing. **Anna Kirwan:** data curation, writing – original draft, writing – review and editing. **Fiona Smyth:** data curation, writing – original draft, writing – review and editing. **Elaine Greene:** supervision, writing – original draft, writing – review and editing.

## Funding

The authors have nothing to report.

## Ethics Statement

This case report was conducted in accordance with the principles of the Declaration of Helsinki. All efforts were made to maintain the patient's privacy and confidentiality.

## Consent

Written informed consent was obtained from the patient for the publication of this case report and any accompanying data or images.

## Conflicts of Interest

The authors declare no conflicts of interest.

## Data Availability

The data that support the findings of this study are available on request from the corresponding author. The data are not publicly available due to privacy or ethical restrictions.
